# Triumph of hope over experience: learning from interventions to reduce avoidable hospital admissions identified through an Academic Health and Social Care Network

**DOI:** 10.1186/1472-6963-12-153

**Published:** 2012-06-10

**Authors:** Victoria Woodhams, Simon de Lusignan, Shakeel Mughal, Graham Head, Safia Debar, Terry Desombre, Sean Hilton, Houda Al Sharifi

**Affiliations:** 1 Department of Health Care Management and Policy, University of Surrey, GUILDFORD, GU2 7XH, UK; 2 Division of Population Health Sciences and Education, Hunter Wing, St. George’s – University of London, LONDON, SW17 0RE, UK; 3 Central Wandsworth Community Ward, Southfield Group Practice, 492a Merton Road, London, SW18 5AE, UK; 4 The Sollis Partnership Ltd 20 Hook Road, Epsom, Surrey, KT19 8TR, UK; 5 Portobello Clinic, 12 Raddington Road, LONDON, W10 5TG, UK; 6 Room 147, 1st Floor, Wandsworth Town Hall, Wandsworth High Street, London, SW18 2PU, UK

**Keywords:** (MeSH): Hospitalization, Health Services Misuse, Outcome and Process Assessment (Health Care), Home Care Services, Hospital based [economics, *organisation and administration]

## Abstract

**Background:**

Internationally health services are facing increasing demands due to new and more expensive health technologies and treatments, coupled with the needs of an ageing population. Reducing avoidable use of expensive secondary care services, especially high cost admissions where no procedure is carried out, has become a focus for the commissioners of healthcare.

**Method:**

We set out to identify, evaluate and share learning about interventions to reduce avoidable hospital admission across a regional Academic Health and Social Care Network (AHSN). We conducted a service evaluation identifying initiatives that had taken place across the AHSN. This comprised a literature review, case studies, and two workshops.

**Results:**

We identified three types of intervention: pre-hospital; within the emergency department (ED); and post-admission evaluation of appropriateness. Pre-hospital interventions included the use of predictive modelling tools (PARR – Patients at risk of readmission and ACG – Adjusted Clinical Groups) sometimes supported by community matrons or virtual wards. GP-advisers and outreach nurses were employed within the ED. The principal post-hoc interventions were the audit of records in primary care or the application of the Appropriateness Evaluation Protocol (AEP) within the admission ward. Overall there was a shortage of independent evaluation and limited evidence that each intervention had an impact on rates of admission.

**Conclusions:**

Despite the frequency and cost of emergency admission there has been little independent evaluation of interventions to reduce avoidable admission. Commissioners of healthcare should consider interventions at all stages of the admission pathway, including regular audit, to ensure admission thresholds don’t change.

## Background

**Figure 1:**
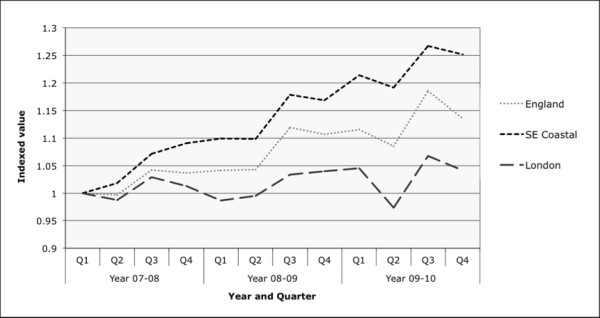
**Relative changes in emergency admission rates across England 2007-2010.** Data collected from the Department of Health QMAE dataset [11] and is presented in year quarters. A relative measure of emergency department admissions has been used by indexing the first quarter 2008 value for each category with a base value of 1. This chart therefore shows the relative changes as opposed to the absolute changes, which enables like-for-like comparison.

Internationally there is growth in hospital use, including unscheduled admissions and this places a cost-burden on health services [1]. This includes the UK, where there has been a relentless year-on-year rise (Figure 1) [2]. The estimated cost of each admission is high; short stay admissions in the UK cost an average of £470, at least double that of an outpatient attendance [3]. Combating this rise in unscheduled care has become a focus of the Department of Health’s Quality, Innovation, Productivity and Prevention (QIPP) programme that aims to make financial efficiency savings whilst maintaining quality of care [4].

Many non-elective admissions have been dubbed ‘avoidable’, ‘inappropriate’ or ‘unnecessary,’ [5-10] and may not reflect patients’ preferences. A considerable proportion are for short stays, often where no procedure is carried out; [11,12] they include ‘high intensity users’ with long-term health conditions admitted with acute exacerbations of their illness. They also include admissions for those with alcohol misuse and psychiatric problems. The top 1 % of health care users in the UK have been estimated to consume 30 % of health care resources [13]. Their admissions are often defined as ‘inappropriate’ because they might be managed using more appropriate (and less expensive) care pathways, particularly lower cost provision in the community [14,15]. Current arrangements are not ideal for doctors or service users. Physicians and patients would prefer around two-thirds of the patients currently managed as emergencies in acute hospitals to be managed in a community setting [10].

Given the 15 % rise in emergency admissions across the UK over the previous three years and marked rates of increase within the local NHS regions (South East Coast 25 % and London 5 % (Figure 1)) [12] the South West London Academic and Social Care Network (AHSN) [16] commissioned this investigation to identify, evaluate and share learning from interventions aiming to reduce ‘avoidable’ admissions in our region.

## Methods

### Overview

Our investigation had three elements: literature review, case study presentations, and two workshops. We used the AHSN as a vehicle for sharing learning and current best practice. We invited people involved in initiatives to reduce unplanned admissions to join this aspect of the Health Outcomes group’s work [17]. We additionally invited all NHS Trusts across the AHSN area, and anyone who had self-nominated themselves or their organisation to be an AHSN member. We also invited those involved in initiatives to identify others who might wish to participate and share their work.

We identified interventions at all stages of the care pathway, putting them in one of three categories: those within the Emergency Department (ED) itself [18], those in primary care aimed at preventing predicted admissions [19-23], and those after the ED evaluating current services [24-26]. We included interventions adopted widely as well as pilot interventions. We also set out to capture the context within which these interventions took place, and understand the importance of context in their success.

### Literature review

We carried out a literature review using Medline using the following search string: “(avoidable OR unnecessary OR inappropriate OR unplanned OR unscheduled OR non-elective) AND (hospital OR accident and emergency OR emergency department) AND admission”. We applied MeSH terms “Hospitalisation”, “Health Services Misuse”, “Home Care Services, Hospital based,” and “Outcome and Process Assessment (Health Care)”.

In addition we searched for specific interventions as keywords: “PARR OR Patients At Risk of Readmission OR Combined Model”; “ACG OR Adjusted Clinical Groups”; “Predictive Modelling”; “Community Matron”; “Virtual Ward”; ”Consultant GP”; “In-reach Nurse OR In-reach Team”; “AEP OR Appropriateness Evaluation Protocol”.

### Data collection

We identified and systematically collected data from schemes introduced to control the rise in inappropriate acute admissions across the AHSN. We used a snowball sampling method asking all respondents to identify any local initiatives they were aware of. We invited those we identified to attend a workshop and describe: (1) The patient group their service was designed to target; (2) The setting of the service; (3) The intervention that the service provided; (4) Any actual or intended outcome measure; and (5) Any other key contextual information.

### Workshop and presentations

The half-day workshops took place in southwest London on 14^th^ January and 20^th^ May 2011. Presenters were asked to provide evaluative data about the use or proposed use of interventions in a standard format: we supplied a template using the five headers listed above. 16 people attended the first, which described seven case studies; and 14 people attended the second, which summarised the findings since the previous meeting and develop a consensus about what interventions might work best and next steps.

### Identifying outcome data: change in local hospital admission rates

We explored whether we could aggregate the data from the studies and investigated whether the interventions reduced hospital admission or ED attendance rates.

### Ethical considerations

As a service evaluation this study did not require full research ethics approval [27]. It utilised reports of innovations in service delivery instigated by NHS managers and was not part of any trial or research process; no individual patient data were presented, nor made available to the researchers.

## Results

### Classification of the type of interventions presented at the workshop

**Figure 2:**
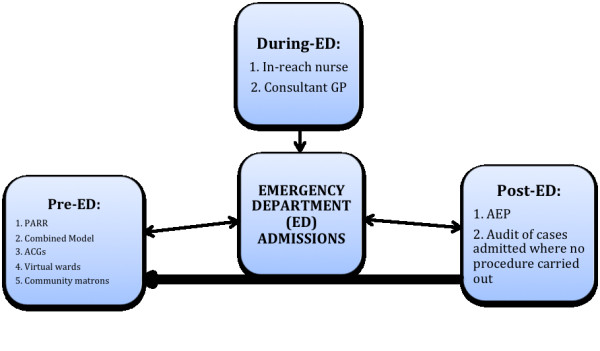
Interventions to reduce avoidable hospital admissions showing interactions between interventions and different stages of the admission pathway.

We identified seven interventions (Table 1), which we divided into three care-pathway stages: pre-, during and post-ED attendance (Figure 2). Pre-attendance interventions included two predictive modelling tools and the deployment of two specialist clinical teams, during ED included two specialist clinical teams, and post-attendance included one clinical audit and feedback intervention (Table 2).

**Table 1:** Summary of the case studies

**Stage**	**Intervention**	**Description**
**Pre-Emergency Department**	**Patient At Risk of Readmission (PARR) tool and Combined Predictive Model**	Tool providing a risk score, which predicts risk of hospitalisation in upcoming 12 months. Uses data from inpatient and census data but Combined Predictive Model can additionally combine outpatient, emergency department and GP practice data. Links data confidentially.
	**Adjusted Clinical Groups predictive model (ACG-PM)**	Predictive modelling tool predicting risk of hospitalisation and where interventions (i.e. active case management) will have the greatest effect. Uses multiple data from primary care, Outpatient and ED data, including demographics, co-morbidities, and prescribing. Whole populations are modelled including non-health care users.
	**Virtual Wards**	Multidisciplinary Team (MDT) managing patients at high predicted risk in their own home with encouragement of self-management. MDT involves GPs, community matrons, ward clerks, district nurses, palliative care, pharmacist, Social Services, etc. Consists of initial assessment, agreed care plan and goals, regular contact and weekly MDT meetings.
	**Community Matrons**	Community-based case management of high-intensity health care users by senior nurses. Often work within the MDT of virtual wards.
**During Emergency Department**	**In-reach nurse**	As part of the Community In-Reach Team (CIRT), in-reach nurses' role is to facilitate discharge, avoid admission, link to community services, and speed investigations for suitable patients in emergency department.
	**Consultant GP**	Consultant GP based in emergency department to identify suitable patients and facilitate discharge. Techniques used include reassurance of staff/patient/family, medication adjustments, liaison with patient's GP, referrals to alternative care pathways, and gaining specialty advice.
**Post-Emergency Department**	**Appropriateness Evaluation Protocol (AEP)**	Validated audit tool used on notes of admitted patients to determine appropriateness of their admission and stay in acute bed. Feedback is then given to improve practice.

**Table 2:** Evaluation of the case studies

**Stage**	**Intervention**	**Description**
**Pre-Emergency Department**	**Patient At Risk of Readmission (PARR) tool and Combined Predictive Model**	Tool providing a risk score, which predicts risk of hospitalisation in upcoming 12 months. Uses data from inpatient and census data but Combined Predictive Model can additionally combine outpatient, emergency department and GP practice data. Links data confidentially.
	**Adjusted Clinical Groups predictive model (ACG-PM)**	Predictive modelling tool predicting risk of hospitalisation and where interventions (i.e. active case management) will have the greatest effect. Uses multiple data from primary care, Outpatient and ED data, including demographics, co-morbidities, and prescribing. Whole populations are modelled including non-health care users.
	**Virtual Wards**	Multidisciplinary Team (MDT) managing patients at high predicted risk in their own home with encouragement of self-management. MDT involves GPs, community matrons, ward clerks, district nurses, palliative care, pharmacist, Social Services, etc. Consists of initial assessment, agreed care plan and goals, regular contact and weekly MDT meetings.
	**Community Matrons**	Community-based case management of high-intensity health care users by senior nurses. Often work within the MDT of virtual wards.
**During Emergency Department**	**In-reach nurse**	As part of the Community In-Reach Team (CIRT), in-reach nurses' role is to facilitate discharge, avoid admission, link to community services, and speed investigations for suitable patients in emergency department.
	**Consultant GP**	Consultant GP based in emergency department to identify suitable patients and facilitate discharge. Techniques used include reassurance of staff/patient/family, medication adjustments, liaison with patient's GP, referrals to alternative care pathways, and gaining specialty advice.
**Post-Emergency Department**	**Appropriateness Evaluation Protocol (AEP)**	Validated audit tool used on notes of admitted patients to determine appropriateness of their admission and stay in acute bed. Feedback is then given to improve practice.

### Pre-emergency department admission interventions: (1) Predictive-modelling tools

Two predictive tools were presented that use routine health data: (1) Patients at Risk of Readmission (PARR) and later developments of this tool; and (2) Adjusted Clinical Groups-Predictive Model (ACG-PM). Both can combine data confidentially to create a ’12-month risk of hospitalisation’ for all patients; although the models have different degrees of sophistication with the ACG-PM having a greater emphasis on co-morbidities. They are used to target interventions, such as community-based case-management, at high-intensity users who consume a disproportionate amount of services. Both have proved easy to integrate and are widely used in practice, with PARR freely available and used by many UK family practices. The ACG-PM is used extensively in the USA but now being made available to UK family practices. They have been extensively validated [1,20-22].

### Pre-emergency department admission interventions: (2) Case management

Two clinical teams were discussed that function ‘upstream’ in primary care, and which case-manage high-risk patients: community matrons, who are specially trained nurses, and virtual wards, which are multidisciplinary teams including the same professions as found on an inpatient ward [23]. Both encourage self-management with regular contact and agreed care plans and goals. They are linked to primary care services, but their capacity limited in relation to target population (i.e. numbers of people admitted with no procedure carried out). However, in our case study they reported that community matrons sometimes had difficulty in filling their caseload. The criteria for taking on cases are generally linked to PARR score; however delays in updates of hospital data and some lack of training may limit PARR’s effectiveness [28].

### Interventions within the emergency department: In-reach nurse and “Consultant GP”

Two clinical teams have been introduced in the ED with the goal of facilitating discharge for those patients amenable to non-hospital mangement [15]. The two examples presented were the in-reach nurse (part of the community in-reach team (CIRT)) and a Consultant GP. Methods employed include speeding investigations, linking to community services and gaining specialist advice. This is a newer concept, with only pilot studies running in the AHSN. They have proved difficult to integrate into ED services.

### Post-admission intervention: Notes audit and Appropriateness Evaluation Protocol (AEP)

The third type of intervention was audit, and the use an audit tool called the Appropriateness Evaluation Protocol (AEP). The former involved the audit of cases where no procedure was carried out; and were admitted less than three days. (i.e. An admission to hospital where no surgical or other procedure is performed.) The rationale is that people who have not any procedure carried out are people who could potentially have been managed in the community. Many people appeared to be admitted for “zero-days” – purely because they were in the ED for over 4-hours, and technically became an admission at that point. The AEP is a type of ‘Utilisation review’ that assesses the appropriateness of acute admissions by retrospective note review across 27 set criteria in order to guide commissioning and operational improvement. This widely validated tool [7,24,25] has only been used in a select pilot in the area, with a sample of 60 patient notes [29].

### Comparing evaluation findings with changes in emergency department admissions in primary hospitals local to the intervention

**Figure 3:**
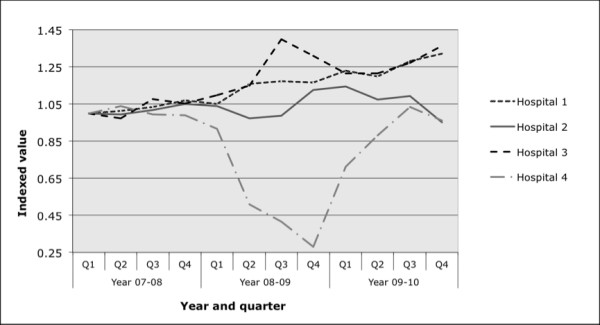
**Relative changes in emergency admission rates for St George’s Hospital, Medway Hospital, Royal Surrey County Hospital (RSCH) and Mayday Hospital March 2007 to April 2008.** St George’s =1, Medway =2, Royal Surrey County Hospital (RSCH) = 3 and Mayday Hospital = 4. Data collected from the Department of Health QMAE dataset [11]. A relative measure of emergency department admissions has been used by indexing the quarter 1 2008 value for each category with a base value of 1.

The evaluation data presented were both qualitative (involving questionnaires) and quantitative (involving cohort studies), only sometimes commenting on the efficacy of the intervention to reduce avoidable admissions. We looked to see if there was any indication that the intervention reduced admission rates, or rate of increase in the ED closest to the intervention or if there were any way of aggregating the data from the interventions (Figure 3). Of the four local hospitals, emergency admission rates for two of the hospitals followed the regional trend of an increase, however there were contrary trends showed by the other two hospitals that were studied; therefore we cannot derive any firm conclusions from these data. The effect of the Wandsworth virtual wards on unplanned hospitalisation rates is currently being evaluated [30].

### Learning from the workshop

The principal lessons from the workshop were how many of the interventions were commissioned by a small number of people driven by the imperative that “something must be done”. Few underwent any evaluative scrutiny, though all were approved through the local NHS governance process, board and/or received local health service director level approval. Many of the presenters described projects they were personally involved in covering areas where they worked. They did not consider bias and did not report that their evaluation might lack objectivity. Only two had considered independent evaluation; and one evaluation was currently underway [30]. Where evaluation was considered generally no funding was available.

The method and scale of evaluations reported varied: five described completed local studies across three main hospital areas; one described a large-scale US study using the same intervention, and one presented multiple background studies from outside the region.

The intervention in all but one locality acted at a single point on the pathway. The one that considered intervening in more than one stage in the pathway combined the use of an in-reach nurse with feedback of data to practices on comparative rates of admission. However, no data were presented about feedback of data.

The presenters came from a wider range of backgrounds and may have only been employed or contracted to the NHS to work on a particular intervention. They included members of private as well NHS healthcare services, self-employed and directly employed clinicians and non-clinicians.

### Post workshop discussion

Between and after the workshops there was some sharing of additional data and research findings. No real consensus emerged other than an unchallenged view that audit was the preferred change mechanism for primary care; and strong views from some individuals that introducing an independent evaluative culture was needed.

## Discussion

### Principal findings

Many of the individual projects claimed success, yet in aggregate they have failed to halt or slow the rise in ED admissions (Figure 3). Current evaluations of interventions are neither independent nor standardised, and do not appear to allow for clustering in their design. Interventions appear to be carried out in isolation; and health service managers do not appear to be considering the impact of multiple interventions at different stages of this care pathway.

### Implications of the findings

Well-meaning interventions are ineffective at a macro level- whether because the relentless rise in admission is too great; the interventions are not sufficiently powerful in isolation, or they are not being used to their full potential. All three are probably contributory.

We need an objective and possibly independently run evaluative protocol including standardised outcome measures more accurately to appraise the effectiveness of different initiatives, enabling the most successful to be identified. They should consider including patient experience. Only one of the studies identified reported patient experience as an outcome measure.

A more integrated “system-based” approach with better teamwork may create improved results; through combined strength and insight into the various interactions initiatives have at different stages of the care pathway.

### Comparison with literature

The increase in ED attendance and non-elective admission is an international problem [1,8,31]. Better community care for long-term conditions can decrease the rate of inappropriate admissions, despite other factors such as social deprivation being outside of healthcare control [9]. Individualised care programmes for patients at high risk of hospitalisation have been effective at reducing health costs in countries including Sweden, Germany, America and the UK [1,8,18,32] and incorporating risk profiling tools into UK-based systems has proved unproblematic [18]. However, there are multiple systems in place (see Table 3); and contradictory findings about their effectiveness; [25,33-35] and some patients at high predicted risk of admission may not be amenable to preventative care [36].

**Table 3:** Predictive modelling tools, clinical teams in primary care and auditing tools for appropriateness of admission in common use

***Predictive modelling tools***	***Primary care-based clinical teams***	***Appropriateness of admission evaluation tools***
-**PARR**	-**Community Matrons**	-**AEP** (including several country-specific versions)
-**PARR++**	-**Virtual Wards**	
-**Combined Predictive Model**	-**Guided Care Model**	-**ISD** - Intensity (of service), Severity (of illness), Discharge criteria, InterQual Inc.
-**ACG**	-**PACE**-Program of All-Inclusive Care for the Elderly	-**MCAP**- Managed Care Appropriateness Programme
-**HUM**-Dr Foster high impact user management tool	**GRACE**- Geriatric Resources for Assessment and Care of Elders	-**MPAP-**Medical Patients Appropriateness programme
-**CPM**-Health dialog Combined Predictive Model		-**The Oxford Bed study instrument**
-**PRISM**-Welsh Predictive Risk Stratification Model		-**SMI-** Standardised Medreview Instrument
-**SPARRA**- Scottish Patients At Risk of Readmission and Admission		-
-**ADRIntell**		-

The use of geriatricians and more senior doctors in the ED has been proposed as yet another mechanism for reducing unnecessary admissions; again these appear to be largely observational studies performed outside of the UK [18,37].

There have been previous attempts to evaluate the use of interventions to prevent avoidable hospital admissions [21] particularly focusing on predictive modelling programmes [18-20] and auditing the appropriateness of admission [7,25,38-40]. These have made useful suggestions, such as greater sensitivity with graduated combination of multiple data sources [19] and looking at the impact of interventions not solely the risk [8]. However, none have taken a whole system perspective.

We found little use of strategies aimed at reducing readmission, beyond the use of risk scores. Reducing readmission is important internationally, and interventions targeted at this part of the care pathway could have an important effect on admission rates [41]. Improved discharge planning [42], exercise programmes with telephone interventions [43], and other interventions may reduce readmission rates. However, systematic reviews failed to find an effect on 30-day readmission [44] or that risk predictive models for readmission are generally poor [45].

Multiple individual interventions may improve outcomes. An integrated-care pilot performed in Torbay [46] has claimed this kind of impact. In this locality health and social services have been linked with newly appointed co-ordinators, extending community support to patients and leading to dramatically reduced rates of avoidable hospital admission.

The Department of Health has recently announced it will not be funding an upgrade of PARR or the Combined Predictive Model encouraging local NHS organisations to “either upgrade [PARR and the Combined Predictive Model] themselves or move to an alternative model” [47].

### Limitations of the method

Case studies were selected from a convenience sample of volunteer attendees at the AHSN. There is a risk of a bias towards the presentation of successful interventions. Case studies were often unpublished, neither peer-reviewed nor independently evaluated. The case studies were of small-scale interventions, incomplete and yet reported some level of success.

### Call for further research

In spite of the limitations, this approach offers an opportunity to bring together an appropriately wide range of healthcare professionals and stakeholders to report current practice. Creating a standard data set embedded into routine practice would provide data and improve our ability to evaluate current interventions.

## Conclusions

This report from an AHSN identified a number of interventions across all stages of the emergency care pathway. A breadth of evidence was presented that illustrated the value of the AHSN for a forum for sharing interventions and for innovators to meet.

However, lack of a fixed evaluative framework meant we were unable fully to compare and contrast the interventions. NHS funds might be better directed to more cost-effective community interventions than to resourcing the apparently ever-rising rate of emergency admissions. Initiatives to combat the latter have, thus far proved ineffective at the macro-level. Methods are more likely to involve co-ordination and possibly integration of services, but only after they have been systematically and independently evaluated. Without mandatory critical appraisal and evaluation of new interventions we will continue to see them introduced more in hope than expectation of success.

## List of abbreviations used

A&E, Accident and Emergency; ACG-PM, Adjusted Clinical Groups Predictive Model; ACG, Adjusted Clinical Group- a predictive modelling tool; AEP, Appropriateness Evaluation Protocol – an audit and feedback tool; AHSN, Academic Health and Social Care Network; AHSN, Academic Health and Social Care Network; CIRT, Community In-Reach Team – a clinical team; CPM, Combined Predictive Model – a predictive modelling tool; ED, Emergency department; GRACE, Geriatric Resources for Assessment and Care of Elders; HUM, High impact User management tool - a predictive modelling tool; ICT, Intermediate care team – a clinical team; ISD, Intensit, Severity, Discharge- an audit tool; MAU, Medical Assessment Unit; MCAP, Managed Care Appropriateness Programme; MPAP, Medical Patients Appropriateness Programme; MDT, Multiple Disciplinary Team; MeSH, Medical Subject Heading; PARR, Patients at Risk of Readmission –a risk score; PACE, Program of All-Inclusive Care for the Elderly; PCT, Primary Care Trust; QIPP, Quality, Intervention, Productivity and Prevention programme; SPARRA, Scottish Patients At Risk of Readmission and Admission..

## Competing interests

VW None declared. SdeL None. Director of South West London AHSN Health Outcomes Group. SD None declared. GH An employee of Sollis – who provide ACGs as a service to GP practices across England. SM None declared. TD None declared. SH None. Co-chair of South West London AHSN Health Outcomes Group. HA-S None. Chair of South West London AHSN Health Outcomes Group.

## Authors’ Contributions

VW Conducted the literature review and major contribution to all drafts of the paper. SdeL Conceived the idea for the learning events and review. Leads the South West London AHSN Health Outcomes Group and helped draft and comment on the paper. SD Contributed to the organisation and content of the first learning event and drafts of the paper. GH Contributed to the content of the first learning event and commented on drafts of the paper. SM Contributed to the content of the first learning event and drafts of the paper. TD Contributed to and commented on all drafts of the paper. SH Contributed to and commented on all drafts of the paper and development of the health outcomes group. HA-S Contributed to and commented on all drafts of the paper and development of the health outcomes group. All authors read and approved the final manuscript.

## Authors’ Information

VW Academic Foundation Doctor Royal Surrey County Hospital. SdeL Professor of Primary Care & Clinical Informatics. SD GP North West London, NIHR In-Practice Fellow. GH Head of Business Intelligence and Data Warehousing, The Sollis Partnership. SM GP Community Consultant Wandsworth PCT. TD Professor of Health Care Management. SH Professor of Primary Care, Head of Division of Population Health Sciences & Education. HA-S Director of Public Health.

## Pre-publication history

The pre-publication history for this paper can be accessed here:

http://www.biomedcentral.com/1472-6963/12/153/prepub
